# Mantises Jump from Smooth Surfaces by Pushing with “Heel” Pads of Their Hind Legs

**DOI:** 10.3390/biomimetics10020069

**Published:** 2025-01-22

**Authors:** Hanns Hagen Goetzke, Malcolm Burrows, Walter Federle

**Affiliations:** Department of Zoology, University of Cambridge, Cambridge CB2 3EJ, UK; hannshagen.goetzke@gmail.com (H.H.G.); mb135@cam.ac.uk (M.B.)

**Keywords:** adhesion, biomechanics, kinematics, jumping insects, take-off

## Abstract

Juvenile mantises can jump towards targets by rapidly extending their middle and hind legs. Here, we investigate how mantises can perform jumps from smooth surfaces such as those found on many plants. *Stagmomantis theophila* mantises possess two distinct types of attachment pads on each foot: three small proximal euplantulae (“heel pads”) with microscopic cuticular ridges and one smooth large distal pair of euplantulae (“toe pad”). Microscopy showed that the surface contact of heel pads is strongly load-dependent; at low normal forces, they make only partial surface contact due to the ridges, but at higher loads they switch to larger areas in full contact. By analysing the kinematics of 64 jumps of 23 third-instar nymphs from glass surfaces and the foot contact areas of their accelerating legs, we show that heel and toe pads fulfil distinct roles. During the acceleration phase of jumps, the contact area of the hind legs’ heel pads tripled, while that of the toe pad decreased strongly, and the toe pad sometimes detached completely before take-off. Although the middle legs also contribute to the jump, they showed a less consistent pattern; the contact areas of their heel and toe pads remained largely unchanged during acceleration. Our findings show that jumping mantises accelerate mainly by pushing with their hind legs and produce grip on smooth surfaces primarily with the heel pads on their proximal tarsus.

## 1. Introduction

Wingless juvenile mantises can jump precisely onto targets to cross gaps between twigs and leaves [1,2,3]. They power their jump with a rapid movement of their middle and hind legs, depressing the trochanter and femur as well as extending the tibia, while the front legs are raised off the surface [2]. Mantises regularly forage on leaves and plant stems, many of which have smooth surfaces. On such surfaces, jumping forward with a low take-off angle is potentially difficult, because the insect has to generate friction forces larger than normal forces. This is only possible if the friction coefficient µ > 1. If one makes the assumption that jumping insects only rely on the classical friction of hard cuticles on the substrate, they could only make upward jumps with take-off angles > 70°, since typical friction coefficients between solids are low (e.g., claws on glass: µ = 0.35, [4]). Insects must therefore improve their foot contact during the acceleration phase to generate sufficiently large friction forces. At the same time, their feet should be able to detach easily at take-off to avoid slowing down. We recently showed that leafhoppers and froghoppers have overcome this biomechanical challenge in two different ways. Leafhoppers (*Aphrodes bicinctus*/*makarovi*) produce the high friction forces required for a jump with several soft, pad-like structures (platellae) on their hind tarsi, which contact the surface only during the acceleration phase of the jump [5]. By contrast, froghoppers (*Philaenus spumarius*) produce high friction when accelerating for a jump by piercing the substrate with sharp spines of their tibia and tarsus [6]. Like froghoppers and leafhoppers, mantises are able to jump from smooth surfaces with low take-off angles. How are their legs able to produce sufficient friction for jumping?

In this study, we investigate how middle and hind legs contribute to jumps of third-instar *Stagmomantis theophila* mantises and what foot structures these insects engage in each leg pair when jumping from smooth surfaces.

## 2. Materials and Methods

*Stagmomantis theophila* (Rehn, 1904) mantises were raised from eggs of five adult males and four adult females and kept in individual boxes at room temperature. We studied the jumps of 23 third-instar nymphs (body mass: 24.8 ± 1.7 mg, mean ± S.D.).

We investigated the tarsus morphology of third- and fourth-instar nymphs using light and scanning electron microscopy. Images of front, middle, and hind feet were taken with a Canon EOS 60D digital camera attached to a Leica MZ16 stereomicroscope (Leica Microsystems GmbH, Wetzlar, Germany). For scanning electron microscopy (SEM), legs of freeze-anesthetised mantises were cut off at the femur, and immediately transferred into fixative (4% glutaraldehyde in 0.1 M PIPES buffer at pH 7.3) for 48 h at 6 °C. Legs were washed in de-ionised water and gradually dehydrated with increasing concentrations of ethanol (final concentration: 96%). Specimens were air-dried, mounted on SEM stubs, and sputter-coated with a 20 nm gold layer. Images were taken with a FEI XL30-FEG SEM (Oxford Instruments, Abingdon, UK) at 5 kV.

### 2.1. Effect of Normal Force on Adhesive Pad Contact Area

A ’see-saw’ lever device was used for observing the effects of normal force on the surface contact of heel pads under the microscope (Figure S1). Two live fourth-instar mantises were mounted on their back on a light plastic sheet attached to one end of a threaded metal rod. One leg was fixed to a thin metal wire glued to the plastic sheet so that either a heel or toe pad were exposed as the highest point. The threaded rod rested on a low friction pivot, and nuts were screwed onto the opposite side of the rod to exactly balance the torque. The pads were brought into contact with a glass coverslip using a micromanipulator. Additional counterweights were then carefully attached to the rod using a micromanipulator to achieve a well-defined increase in normal load. Contact areas were imaged using a 5× or 100× oil immersion objective and monochromatic (546 nm) epi-illumination with a QICAM 10-bit monochrome camera (Qimaging, Burnaby, BC, Canada) mounted on a Leica DMR-HC microscope.

### 2.2. High-Speed Contact-Area Recordings of Jumps

Third-instar *Stagmomantis theophila* nymphs were placed on a glass coverslip on a Leica DM IRE2 inverted microscope and a paintbrush was presented as a target about two body lengths away from the glass coverslip at level height (Figure S2). Moving the paintbrush attracted the mantises’ attention; they mostly walked towards the edge of the coverslip and jumped onto the paintbrush. When the insects jumped from the right position, one foot was visible from below. Only jumps in which the whole tarsus of the recorded leg was on the glass coverslip and did not protrude over the edge were included in the analysis. Two or three cameras recorded 64 jumps of 23 animals from glass coverslips. Two synchronised Phantom V7.1 high-speed cameras (Vision Research, Wayne, NJ, USA) simultaneously captured the jumps with a frame rate of 4700 frames per second, both from the side and from below, the latter using the inverted microscope with a 5× lens and epi-illumination to record contact areas. For 40 of these jumps, a third synchronised high-speed camera (Optronis CR5000x2, Optronis GmbH, Kehl, Germany) was available to capture the jump trajectory from above. Leg detachment was defined as the first frame in which the leg that was visible from below had detached from the surface and this time was defined as t = 0 ms. The first visible movement of middle or hind legs before a jump was taken as the start of the acceleration phase. In most recordings, not all legs were in focus or visible in side view, and it was therefore impossible to determine precisely when the other legs detached. On average, middle legs detached earlier than hind legs, leading to a different mean start time of the acceleration phase for recordings in which middle or hind legs were visible from below.

Contact areas and foot orientation (in the horizontal plane) were measured from the videos. The contact area of the toe pad, and the combined contact area of the three heel pads (i.e., including gaps between the cuticular ridges) were measured using a threshold algorithm in MATLAB (The Mathworks, Natick, MA, USA). The insect’s take-off direction and azimuth (the horizontal angle between the take-off direction and the body orientation at the start of the acceleration phase) was measured from the dorsal view by digitising a point on the thorax at the start of the acceleration phase and when airborne. The foot orientation at the start of the acceleration phase was measured using the midline of the two most distal pads and converted into foot orientation relative to take-off direction. An estimate of the insect’s take-off angle was measured from the side view of 12 jumps by digitising the position of the middle leg coxa at take-off and 2.1 ms after take-off. The digitisation was repeated three times, and the mean was taken to reduce digitising errors. To assess the force vector of middle legs at detachment, we recorded the movement direction of the detaching foot in the initial frames after take-off.

Third-instar mantises were also encouraged with a paintbrush to walk over the glass coverslip on the inverted microscope. Simultaneous contact area and side views of 17 steps of front, middle, and hind legs of six animals were recorded at 800 frames per second.

To determine the extension and acceleration distance of each leg pair during the acceleration phase and the timing of detachment, we analysed 27 jumps of nine third-instar mantises from high-density foam (Plastazote, Watkins and Doncaster, Cranbrook, UK). This dataset was recorded from side views of jumps using one Photron Fastcam SA3 high-speed camera (Photron (Europe) Ltd., West Wycombe, UK) filming at 1000 frames per second. To determine leg extension, the distance between the tibia-tarsus joint and the anterior edge of the coxa was measured at the start of the acceleration phase and in the last frame before take-off. The acceleration distance of middle and hind legs was defined as the distance that the anterior edge of the coxa travelled during the acceleration phase while the leg was in surface contact. Take-off was defined as the first frame in which all legs had detached from the surface and the animal was airborne.

The data were analysed statistically using R v3.0.2 (R Core Team, 2014). Unless specified otherwise, data are presented as means ± standard error of the mean (s.e.m.).

## 3. Results

### 3.1. Morphology

**Figure 1:**
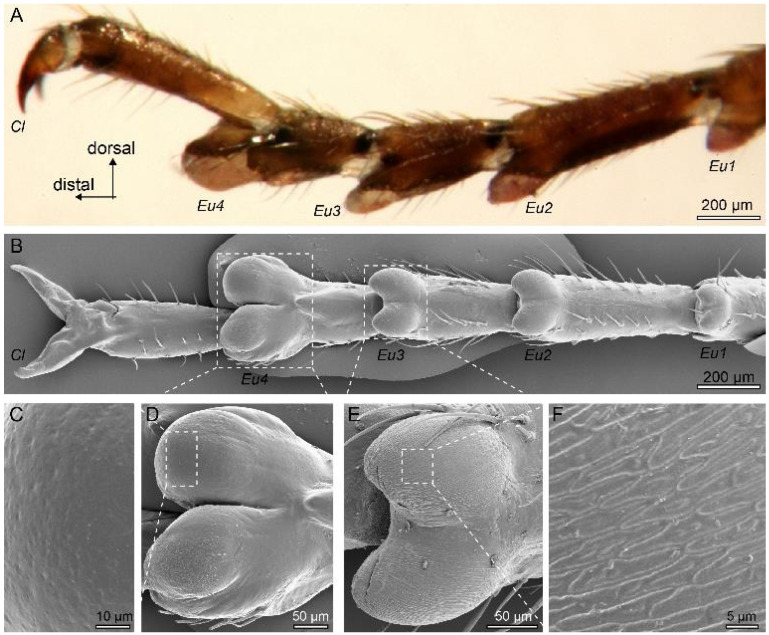
Light and scanning electron microscopy images of hind (**A**, lateral view) and middle legs (**B–F**, ventral view) of a fourth-instar *Stagmomantis theophila* nymph. The tarsi have five segments and euplantulae are located at the distal end of the first four tarsomeres (**A**,**B**). Euplantulae are whitish and less sclerotised than the surrounding cuticle. The most distal pair of euplantulae (Eu4, “toe” pad) have a smooth surface (**C**,**D**) and are larger than the three proximal euplantulae (Eu1-Eu3, “heel” pads), which possess a surface pattern of branching ridges (**E**,**F**). Cl: claws.

The tarsi of all legs of *Stagmomantis theophila* mantises have five segments (tarsomeres). There are attachment pads (euplantulae) ventrally at the distal end of each of the first four tarsomeres. The pads are whitish, softer to the touch, and hence less sclerotised than the surrounding cuticle (Figure 1). The four attachment pads have two distinct designs (Figure 1). The most distal pair of euplantulae (“toe pad”) on the fourth tarsomere has a smooth surface structure (Figure 1). Its projected pad area was 14,920 ± 739 μm^2^, more than twice the size of the pad on the third tarsomere (7179 ± 792 μm^2^, N = 3 third-instars). The three proximal pairs of euplantulae (“heel pads”) increase in size from the first to the third tarsomere (ANOVA: F_1,7_ = 29.7, *p* < 0.001). Their surface consists of a branched pattern of ridges likely formed by the epicuticle (Figure 1E,F). The spacing between the ridges was 1.3 ± 0.1 μm (30 measurements from 10 pads of three animals). No differences of the pads were observed between front, middle, and hind legs.

### 3.2. Load Dependence of Heel Pads

**Figure 2:**
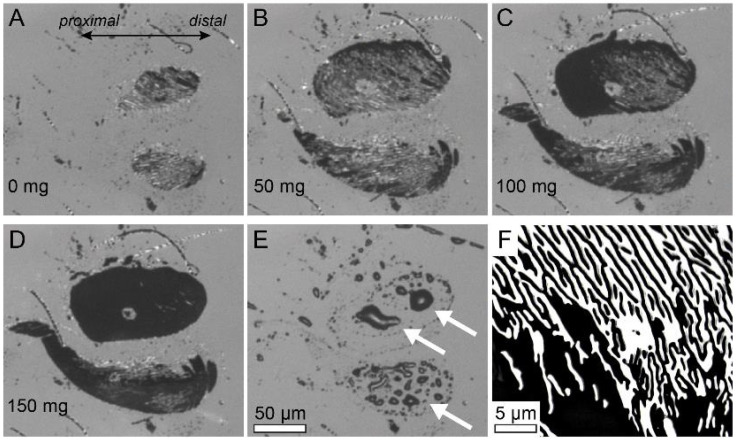
Contact areas of *S. theophila* heel pads (Eu2–Eu3) at different normal loads. Contact area increased with normal load and fluid accumulated in the contact zone (**A**–**D**) and fluid accumulated in the contact zone. Fluid droplets (white arrows) remained on the glass coverslip after removing the pad (**E**). Ridges in surface contact separated by channels filled with air or fluid secretion were visible at high magnification ((**F**), 150 mg load, contrast enhanced for clarity).

The contact areas of the heel pads increased with normal load (Figure 2A–D). At low normal forces, contact areas were small and pads were only in partial contact, i.e., only the ridges but not the channels in between them touched the surface (Figure 2F). At higher normal forces, contact areas increased and pads made full contact with both the ridges and the channels in between them in surface contact, aided by liquid secretion. When the normal load was decreased again, the pads initially remained in full contact. When the leg was detached, fluid droplets were left on the glass coverslip (Figure 2E). For toe pads, the contact areas also increased with load, but full contact already occurred at small normal loads.

### 3.3. Kinematics and Tarsal Contact During Take-Off

**Figure 3:**
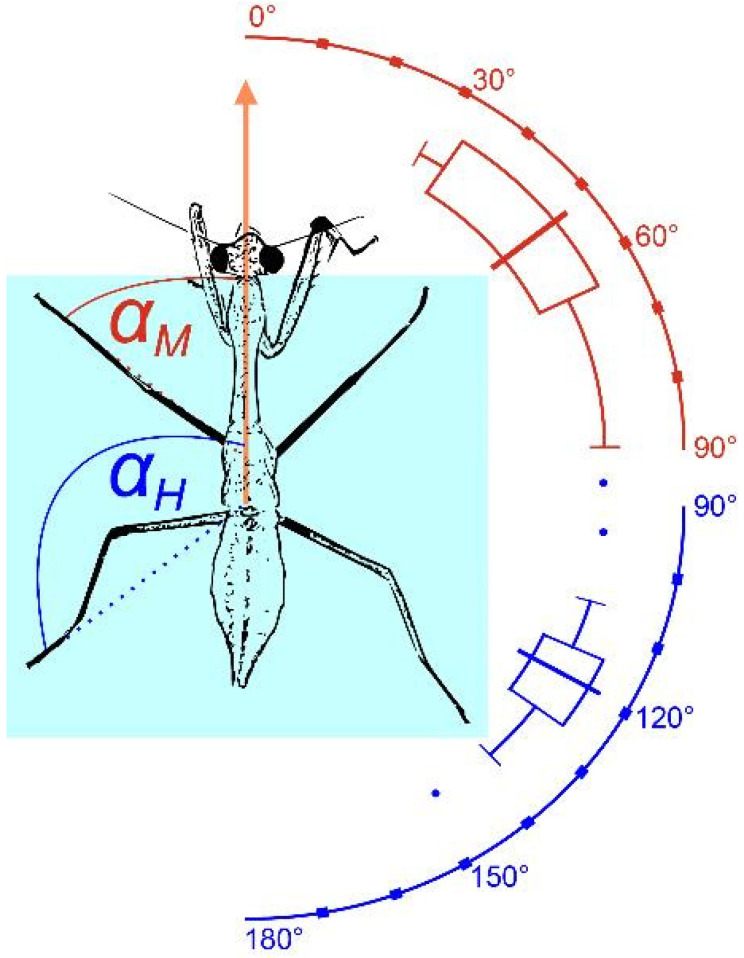
Foot orientation of *S. theophila* hind and middle legs (α_H_, α_M_) relative to the take-off direction at the start of the acceleration phase. When preparing for the jump, mantises placed their forward-pointing middle legs close to the edge of the glass coverslip (light blue) while their hind legs were pointing laterally backward. Front legs are not in contact with the surface during the acceleration phase.

All mantises were able to jump from the smooth glass coverslip without any slipping (n = 64 jumps of 23 insects). Mantises jumped with a mean take-off angle of 7.8 ± 3.1° (range: −10.4 to 27.8°, including downward jumps in which the target was below the glass coverslip). Since the insects jumped towards a target, this angle likely depended on the position of the target offered. At the start of the acceleration phase, the hind legs pointed backwards (mean angle 118.9 ± 2.8°), and the middle legs pointed forwards (mean angle 52.9 ± 4.3°). The front legs were never in contact during the acceleration phase (Figure 3).

**Figure 4:**
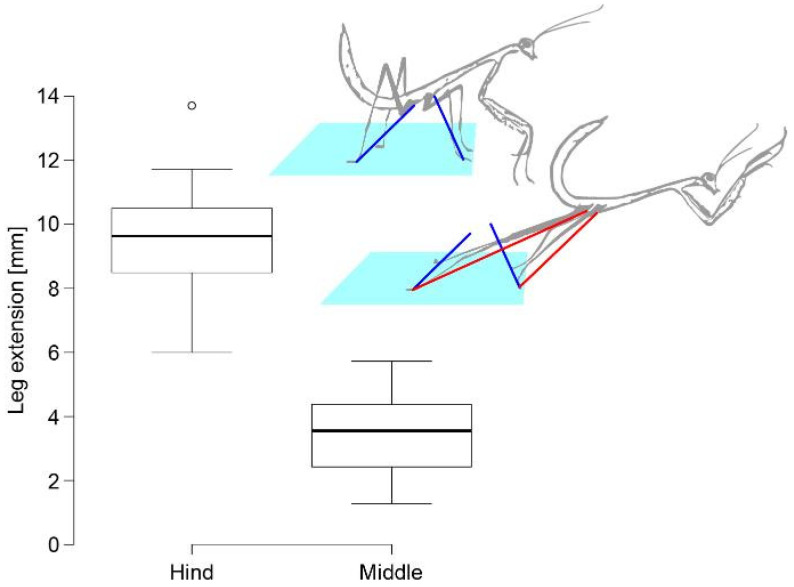
Leg extension of *S. theophila* middle and hind legs during the acceleration phase of a jump. Leg extension was measured as the change in tarsus–coxa distance from the start (blue lines) to the end (red lines) of the acceleration phase.

During the acceleration phase, the hind legs extended significantly further than the middle legs (increase in coxa–tarsus distance: hind legs 9.52 ± 0.33 mm, middle legs 3.42 ± 0.24 mm, 27 jumps by nine third-instar nymphs; Welch’s *t*-test: t_47.1_ = 14.9, *p* < 0.001; Figure 4). At take-off, the middle and hind legs were fully extended. While the hind legs mainly extended in the direction of the jump, the middle legs changed from a forward to a backward orientation by a rotation in the coxa. On average, the middle legs detached earlier than the hind legs (paired *t*-test: t_26_ = 3.13, *p* = 0.004, Figure S3A). The total acceleration distance, i.e., the distance the coxa travelled while the foot was in contact with the surface, was therefore significantly larger for the hind legs than for the middle legs (hind legs 10.30 ± 0.38 mm, middle legs 9.18 ± 0.41 mm; Welch’s *t*-test: t_51.7_ = 2.0, *p* = 0.0498; Figure S3B).

**Figure 5:**
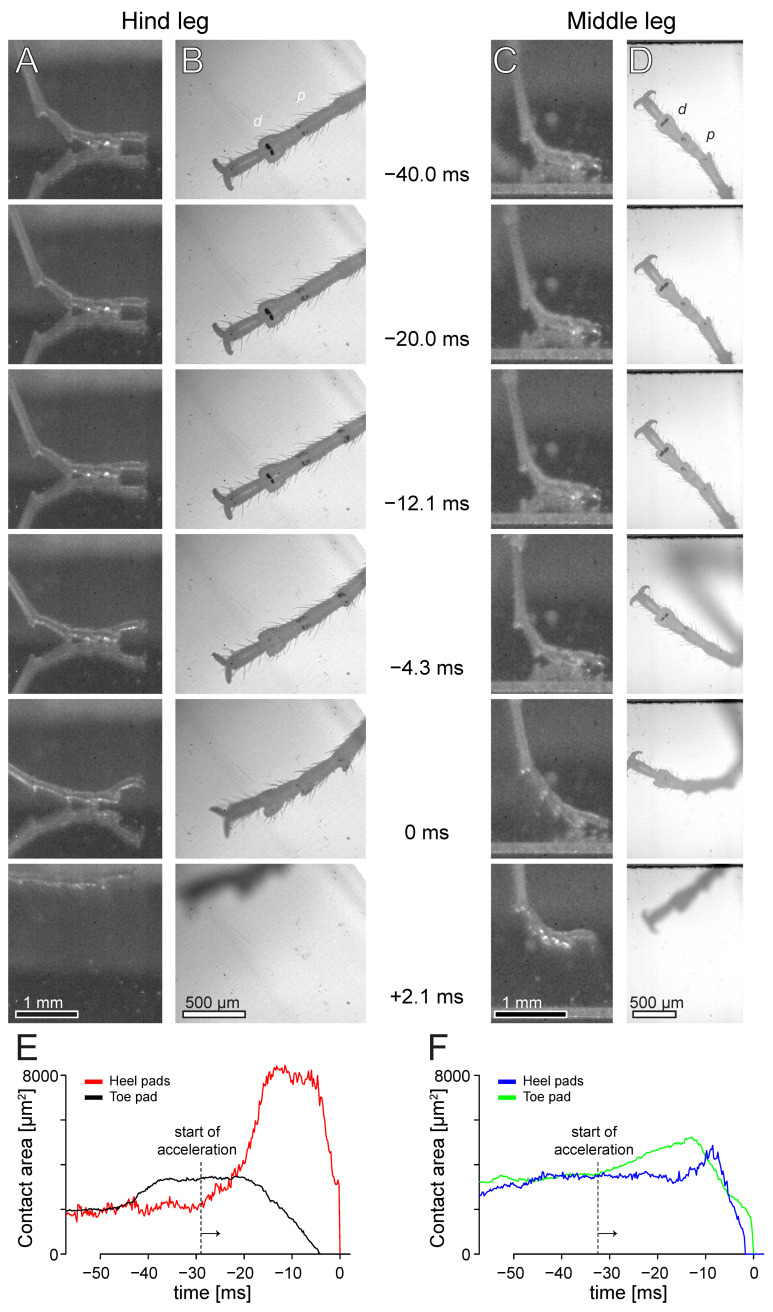
High-speed image sequences of the acceleration phase of two jumps of *S. theophila*, showing hind (**A**,**B**) and middle legs (**C**,**D**) from the side and below, as well as the adhesive contact areas of their heel and toe pads (**E**,**F**). Contact area images are rotated so that the jump direction is toward the top of the page. During the acceleration phase of the jump shown in (**A**,**B**,**E**), which lasted 29.1 ms, the contact area of the hind legs’ heel pads increased fourfold while their toe pads decreased in contact area and detached first. During the acceleration phase of the jump shown in (**C**,**D**,**F**), which lasted 32.3 ms, the contact area of the middle legs’ heel pads increased only slightly and their toe pad did not detach before the heel pads. The first frame in which a propulsive movement of the leg was visible in the side view was defined as the start of the acceleration phase. The first frame without any adhesive contact was defined as detachment and set to 0 ms.

**Figure 6:**
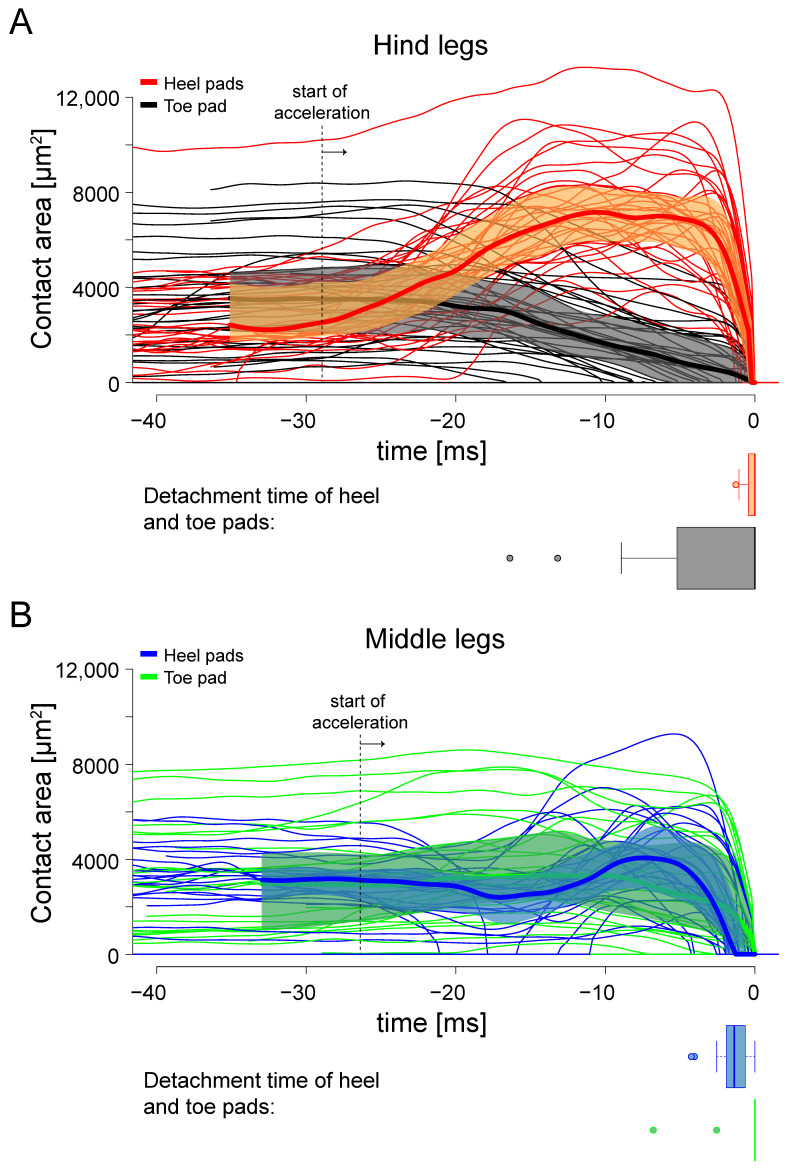
Contact areas for heel and toe pads of hind legs (**A**) and middle legs (**B**) during the acceleration phase of *S. theophila* jumps. Raw data was filtered using a low-pass Butterworth filter with a cut-off frequency of 470 Hz. A median curve (bold line) and interquartile range (shaded) was calculated from 38 jumps by 23 animals for hind legs and 26 jumps by 15 animals for middle legs and plotted for the range of the shortest recorded jump. Horizontal boxplots below the contact area curves indicate the detachment times of heel and toe pads (0 ms is defined as the last pad detaching at take-off). In the hind legs, the changes in contact area differed significantly between heel and toe pads, whereas no clear pattern was visible for the middle legs. The mean start time of the acceleration phase for hind and middle legs is indicated by the dotted line; the time at which the leg detached was set to 0 ms.

We observed a characteristic foot movement and change in surface contact for all hind legs during the jumps (38 jumps by 23 mantises; Figure 5 and Figure 6, Videos S1–S4). While in most jumps, both heel and toe pads came into surface contact, the last two tarsal segments were raised during the acceleration phase and the contact area of the toe pad gradually decreased. In contrast, the projected contact area of the heel pads (both Eu3 and Eu2 in all 38 jumps; in addition, Eu1 came into contact with the surface during only one of the 38 jumps) increased threefold at the start of the acceleration phase and reached a plateau. Although the resolution of the contact area recordings was not sufficient to see the cuticular ridges, the contact zones of the heel pads appeared mostly grey and lighter than those of the toe pads (Figure 5B, Video S2), indicating that they were in partial contact. During the acceleration phase, the contact area of the heel pads often became locally darker, indicating that they made full contact in these regions. In the jumps where both pad types of the hind leg were in contact, the toe pad detached on average earlier than the heel pads (median difference 0.1 ms, mean difference 2.3 ms; Wilcoxon signed rank test: W = 191.5, n= 36, *p* = 0.044; Figure 6). In freely walking mantises, no such pattern for hind leg contacts was observed. Walking mantises used all three leg pairs and both heel and toe pads (Figure S4).

In contrast to the situation in hind legs, we did not observe any lifting of the last two tarsal segments in the middle legs, or any decrease in the contact area of the toe pad or increase in area of the heel pads (26 jumps by 15 mantises; Figure 5 and Figure 6, Videos S3 and S4). The heel pads (Eu3 in all 26 jumps; Eu2 in 17 out of 26 jumps) detached first and lost contact up to 8.1 ms before the toe pad (median difference 1.5 ms, mean difference 1.1 ms; Wilcoxon signed rank test: W(25) = 255.5, *p* = 0.003; boxplots in Figure 6). The contact areas of middle legs varied strongly between jumps. Before the start of the acceleration phase, contact areas of toe and heel pads were similar in both the middle and hind legs (F_3,122_ = 0.8, *p* = 0.481). During the acceleration phase, the contact area of heel pads exceeded that of toe pads only in the hind legs but not in the middle legs. The middle legs often rotated around their foot contact during the acceleration phase and heel pads sometimes detached and re-attached again. Some of the variation in contact area in middle leg toe pads may be explained by the foot orientation prior to the jump: when middle legs were oriented forward, parallel to the jump direction, the maximum contact area of the toe pad was larger. This indicates that the middle legs contributed to the jump acceleration by pulling, and that this increased the contact area most strongly when the legs pointed in the direction of the jump; however, the effect was small (F_1,16_ = 5.3, *p* = 0.035, R^2^ = 0.201).

## 4. Discussion

Our results show that juvenile mantises are able to perform jumps from smooth surfaces without slipping. Contact area recordings during the acceleration phase revealed that they mainly engage the heel pads of their hind legs, suggesting a division of labour between heel and toe pads similar to that found in other climbing insects [7,8,9,10]. Our results suggest that middle and hind legs perform different functions during the acceleration phase. While the hind legs likely generate most of the propulsion, the middle legs can be used to control the jump trajectory.

### 4.1. Division of Labour Between Attachment Pads During the Acceleration Phase of Jumps

As the tarsi of *Stagmomantis theophila* mantises have the same number of attachment pads on the front, middle, and hind legs, and pads are similar in morphology and size, their foot attachment structures are probably not particularly specialised for jumping. Like other mantises, they possess two distinct types of attachment pads: one large pair of euplantulae with a smooth surface on the fourth tarsomere (toe pad) and three smaller pairs of euplantulae with a cuticular ridge pattern on the first three tarsomeres (heel pads). Our observations suggest that the cuticular ridges on the heel pads enable load-dependent control of contact area and thus high friction coefficients combined with low detachment forces, similar to the function of “nubby” stick insect tarsal friction pads with conical cuticular outgrowths [8,11]. The cuticular ridges on the mantises’ heel pads are similar to those reported for *Nauphoeta cinerea* cockroaches, where they have been found to increase friction on rough surfaces [12]. It is possible that the ridges on the heel pads also aid mantises in interlocking on rough surfaces.

Like other mantises [13,14], *Stagmomantis theophila* lack an adhesive pad on the pretarsus, and the distal pair of euplantulae may have taken over the function of the adhesive pad. A similar arrangement and division of labour between two distinct tarsal pad types has been described for *Tettigonia viridissima* and *Acanthoproctus diadematus* bush-crickets [9,15]. For various insects it has been shown that toe and heel pads perform different tasks during climbing; toe pads are mainly used to generate adhesion and friction forces in the pulling direction, whereas heel pads are used to generate friction forces under compression in the pushing direction [7,8,9,10,16]. Jumping mantises used their toe and heel pads in accordance with this division of labour. To enable forward jumps on smooth surfaces, hind legs must generate large friction forces when pushing. As we did not observe any slipping even when mantises jumped from glass with low take-off angles, their pads indeed produced friction forces much greater than the normal forces. As the contact area of the hind legs’ heel pads increased threefold during the acceleration phase, it is likely that these pads are mainly responsible for the high friction. In contrast, the contact area of the hind legs’ toe pads decreased during the acceleration phase, and on average they detached earlier than the heel pads. This suggests that toe pads did not contribute much to the hind legs’ pushing forces, consistent with findings for distal adhesive pads in other insects, which typically detach when pushed [8,10,17,18]. When the toe pads did not detach before the heel pads, their contact areas were very small just before the jump, so that their adhesion could hardly slow down the jump. The middle legs pointed in the jump direction or were oriented laterally to it, allowing them to contribute to the jump by pulling. In contrast to the hind legs, pad contact areas of the middle legs varied strongly between different animals and jumps, but on average did not change much during the acceleration phase. When the tarsi of middle legs were aligned with the jump direction, the maximum contact area of the toe pads was larger, indicating that the middle legs contribute to the jump by pulling.

### 4.2. Contribution of Middle and Hind Legs to the Jump

While the fastest jumping insects only use their hind legs to power their jumps [19,20,21], some insects and jumping spiders accelerate with two leg pairs [22,23,24,25,26,27,28,29,30,31,32]. Three hypotheses have been suggested as to why the use of an additional leg pair might be advantageous [24]: First, spreading the forces over four legs would reduce the force on each individual foot, thereby allowing jumps from softer substrates [24,25]. Second, species with thin legs might require two leg pairs to produce sufficient power to jump [26,28]. Third, two leg pairs might enable the animal to control pitch more easily [24]. Why is it beneficial for mantises to use four legs for jumping?

Juvenile mantises can jump precisely onto targets [1,2]. Similarly, jumping spiders catch prey by jumping with their third and fourth leg pairs while the anterior two leg pairs are lifted off the surface [31]. The spiders’ third and fourth legs can vary considerably in length between species, and thus in their contribution to acceleration [30,33]. If more than two legs contribute to the jump, the different legs could take on different tasks during the acceleration phase. Our results indicate that this is the case for mantises: the different contact area progressions of pads in middle and hind legs, and the larger acceleration distance for hind legs, suggest that hind legs provide most of the thrust for the jump. The contribution of each leg to the kinetic energy of the jump is the integral of that leg’s ground reaction force over the acceleration distance. This distance was significantly larger for the hind legs, as the middle legs usually detached first. If middle and hind legs contributed equally to the energy of the jump, the forces of the middle legs would therefore have to exceed those of the hind legs. However, larger normal forces would probably result in larger contact areas of the heel pads, just as we observed in the hind legs during the acceleration phase. As the contact areas of middle legs were smaller and did not increase much during the acceleration phase, the middle legs may contribute only little to the acceleration of the jump.

**Figure 7:**
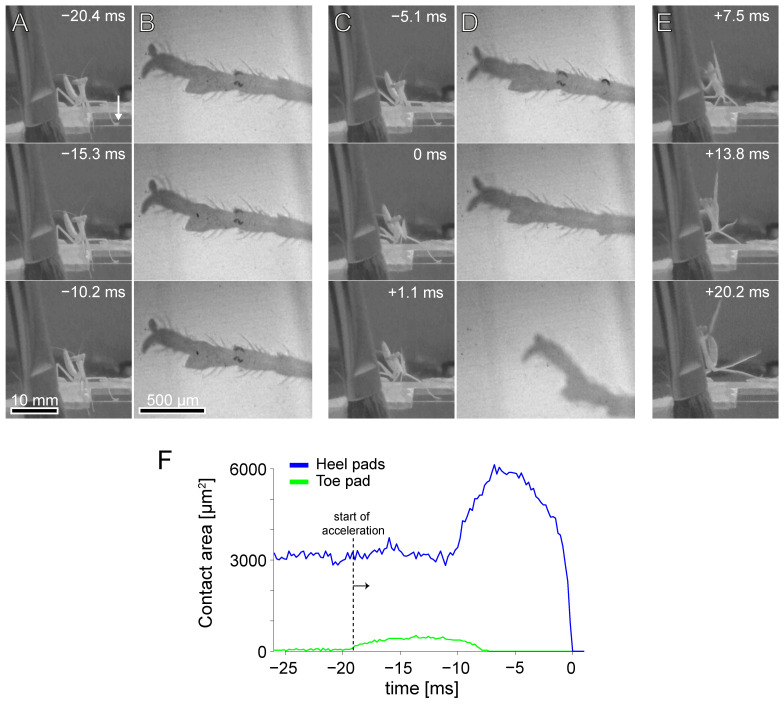
Image sequence of a *S. theophila* jump with a large azimuth angle of 42.1° from the side (**A**,**C**,**E**) and below, showing the left middle leg (**B**,**D**). The adhesive contact area of toe and heel pads of the left middle leg is plotted below (**F**). In (**A**), a white arrow points at left middle leg in the first image. The contact area images are rotated so that the direction of the jump points to the top of the page. In contrast to most other recordings of middle legs, the contact area of the toe pad was very small, and the toe pad detached before the heel pads.

What then is the function of the middle legs for the jump? It is possible that they are mainly used to control the take-off angle, pitch, and azimuth during the acceleration phase of the jump. The control of these parameters has been studied in insects that jump only with hind legs. Locusts adjust the position of their hind legs before accelerating to control their take-off angle [34] and froghoppers adjust the lateral position of their hind legs to control azimuth [35]. Rapidly jumping small insects employ powerful catapult mechanisms, e.g., [19,20,36,37], where the acceleration lasts no longer than a few milliseconds and is therefore too short for neuronal feedback. The jumps of these insects mainly serve a quick escape and are less optimised for a precise landing. In contrast, mantises take much longer to accelerate (juvenile mantises: >20 ms [2,3]), which would in principle allow neuronal feedback to adjust the take-off angle during the acceleration phase. Our observations indicate that middle legs are indeed involved in the control of azimuth: in one jump with an unusually large (right) azimuth, the weighting of heel and toe pads was strongly asymmetrical in the laterally oriented left middle leg and similar to that of the hind legs, indicating that this middle leg pushed sideways to correct the azimuth (Figure 7, Videos S5 and S6). For the targeted jumps of mantises, accurate landing is probably more important than power. Therefore, the additional control by the middle legs and the longer acceleration time may be required for the precise control of take-off to land in the right spot.

## Data Availability

All data of the recorded contact areas and jump kinematics underlying Figure 3, Figure 4, Figure 5, Figure 6, Figure 7, Figures S3 and S4 have been uploaded to the Dryad digital repository (https://doi.org/10.5061/dryad.x69p8czvh, accessed on 14 January 2025).

## Supplementary Materials

The following supporting information can be downloaded at https://www.mdpi.com/article/10.3390/biomimetics10020069/s1. Figure S1: ’See-saw’ setup to visualise the effect of normal load. Sketch of ’see-saw’ setup used to visualise the effect of normal load on the contact area of mantis pads. Mantises were mounted on a plastic sheet and immobilised using Parafilm. One leg was attached using dental wax to a metal wire fixed to the plastic sheet so that the ventral side of one tarsal attachment pad was the highest point. The plastic sheet was attached to a threaded metal cylinder resting on a low friction pivot and was balanced by a nut that acted as a counterweight. Additional weights were placed to achieve defined normal loads. Contact areas were recorded through a microscope using reflected light. Figure S2: Setup to record contact areas. Setup used to record contact areas of mantis attachment pads during the acceleration phase of jumps. Mantises were motivated to jump from a glass coverslip towards a target. The glass coverslip was positioned on an inverted microscope and contact areas were recorded with a high-speed camera using reflected light. The movement of the foot and whole-body movements were filmed from the side and, for some jumps, from above. Figure S3: Relative detachment time of hind vs. middle legs. (A) When jumping, the middle legs detached on average before the hind legs. (B) Acceleration distance (distance travelled by the coxa while the foot was in surface contact) in middle and hind legs. Figure S4: Contact area progression during walking steps. contact area progression during steps of front, middle, and hind legs in walking mantises (front legs: three steps of three insects; middle legs: seven steps of six insects; hind legs: six steps of six insects). Video S1: Mantis jump hind leg side view. High-speed video of a jump of *S. theophila* from a glass surface, showing the left hind leg during the acceleration phase from the side (frames of this video are shown in Figure 5A). The video was recorded at 4700 fps, and the width of the field of view is 9.43 mm. Video S2: Mantis jump hind leg contact area. High-speed video of the same jump of *S. theophila* as in Video S1, showing the left hind leg during the acceleration phase from below (frames of this video are shown in Figure 5B). The video was recorded at 4700 fps, and the width of the field of view is 1.66 mm. Video S3: Mantis jump middle leg side view. High-speed video of a jump of *S. theophila* from a glass surface, showing the left middle leg during the acceleration phase from the side (frames of this video are shown in Figure 5C). The video was recorded at 4700 fps, and the width of the field of view is 10.6 mm. Video S4: Mantis jump middle leg contact area. High-speed video of the same jump of *S. theophila* as in Video S3, showing the left middle leg during the acceleration phase from below (frames of this video are shown in Figure 5D). The video was recorded at 4700 fps, and the width of the field of view is 1.61 mm. Video S5: Mantis jump large azimuth middle leg side view. High-speed video of a jump of *S. theophila* with a large azimuth angle, showing the left middle leg during the acceleration phase from the side (frames of this video are shown in Figure 7A,C,E). The video was recorded at 4700 fps, and the width of the field of view is 21.3 mm. Video S6: Mantis jump large azimuth middle leg contact area. High-speed video of the same jump of *S. theophila* as in Video S5, showing the left middle leg during the acceleration phase from below (frames of this video are shown in Figure 7B,D). The video was recorded at 4700 fps, and the width of the field of view is 1.56 mm.
