# Supplementary figures and images for: Mantises Jump from Smooth Surfaces by Pushing with “Heel” Pads of Their Hind Legs
Source: Biomimetics (Basel). 2025 Jan 22;10(2):69. doi: 10.3390/biomimetics10020069 (PMC11852473; doi:10.3390/biomimetics10020069)

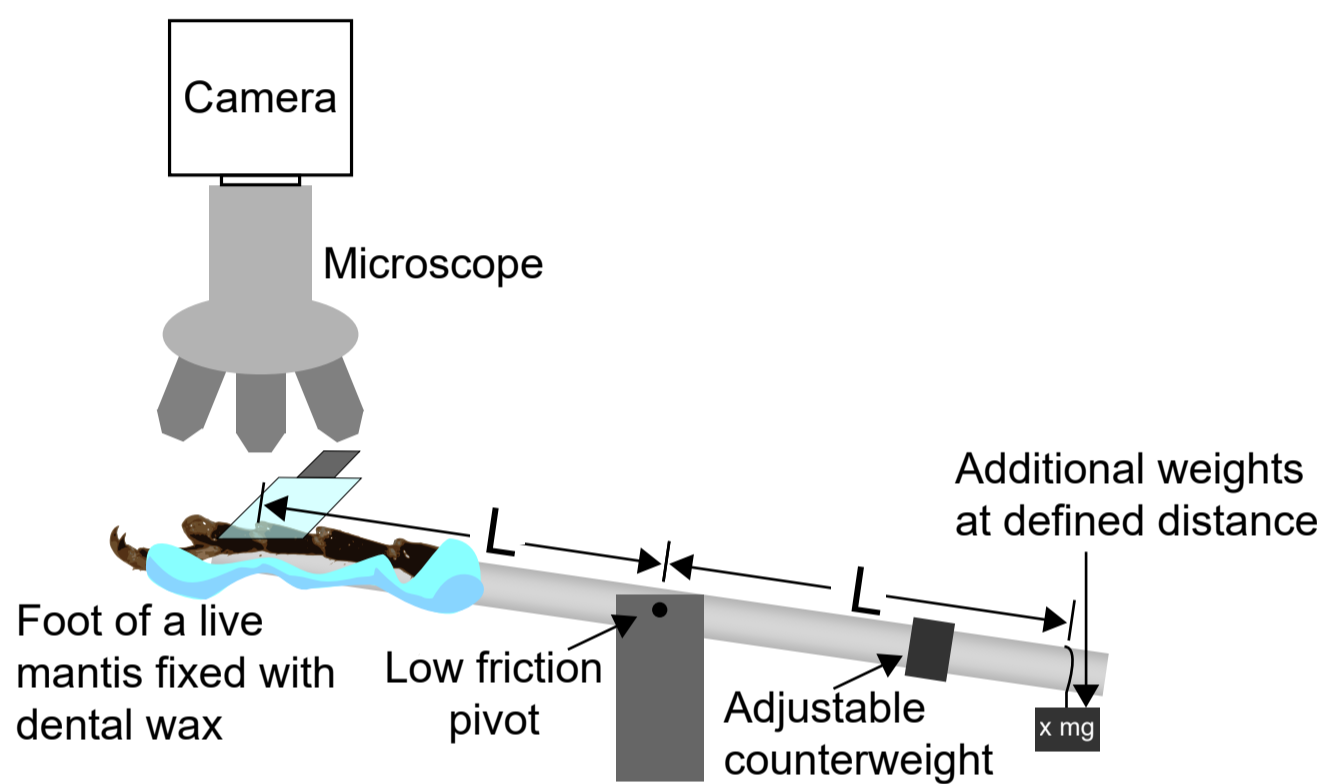

Supplement: Supplementary file 1 [file biomimetics-10-00069-s001.zip › FigS1.pdf]

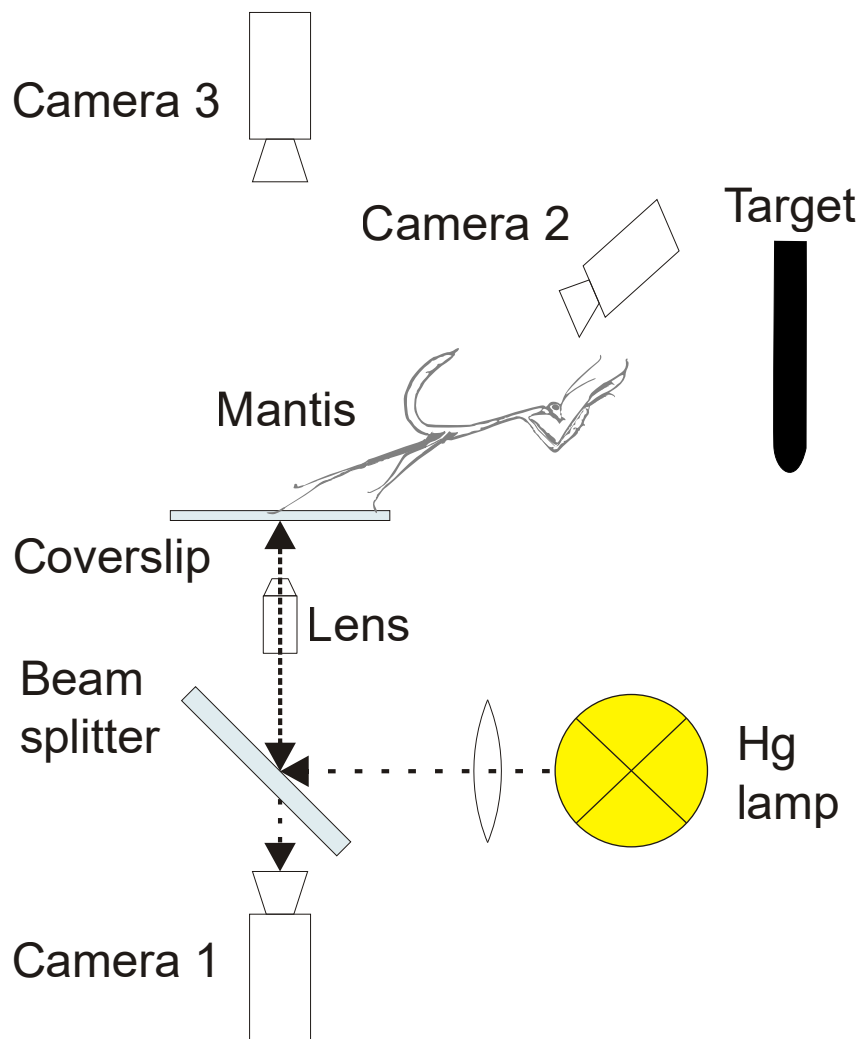

Supplement: Supplementary file 1 [file biomimetics-10-00069-s001.zip › FigS2.pdf]

**A**

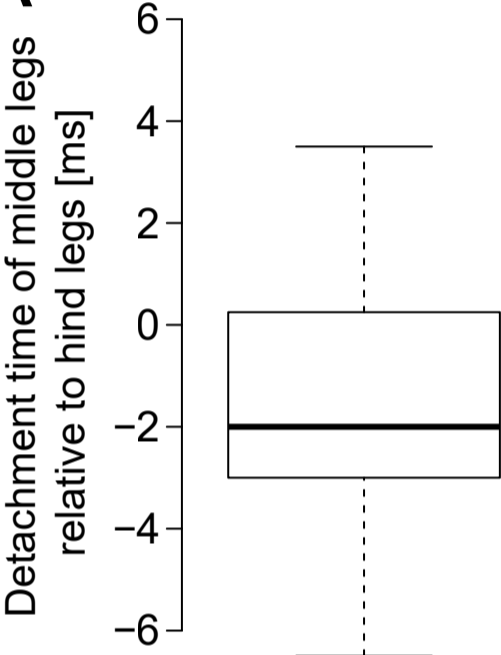

**B**

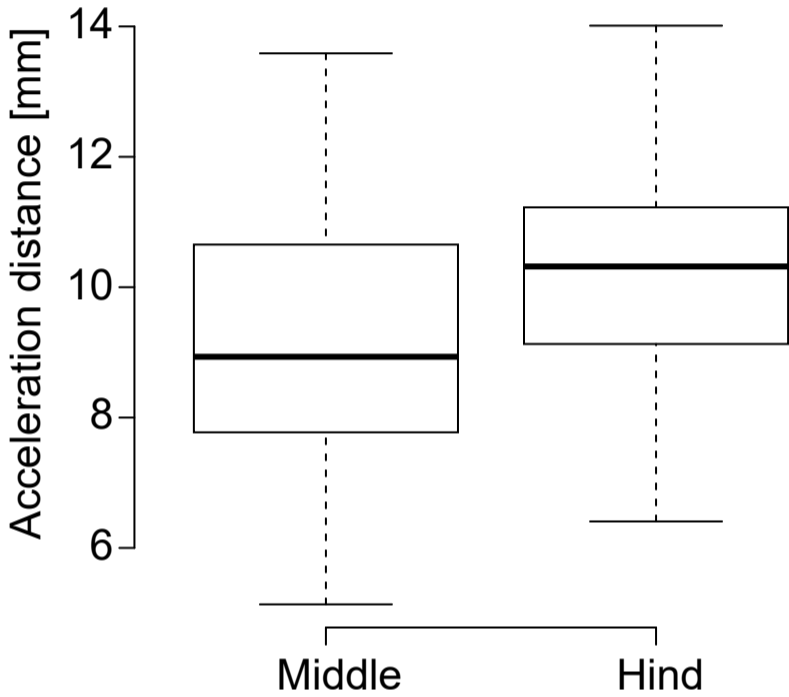

Supplement: Supplementary file 1 [file biomimetics-10-00069-s001.zip › FigS3-rev.pdf]

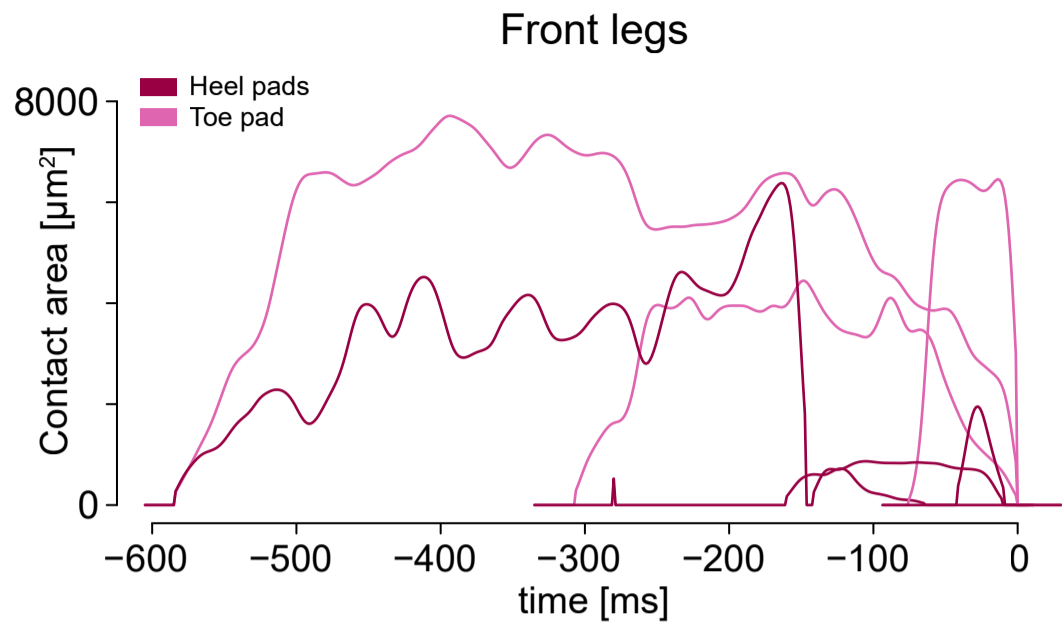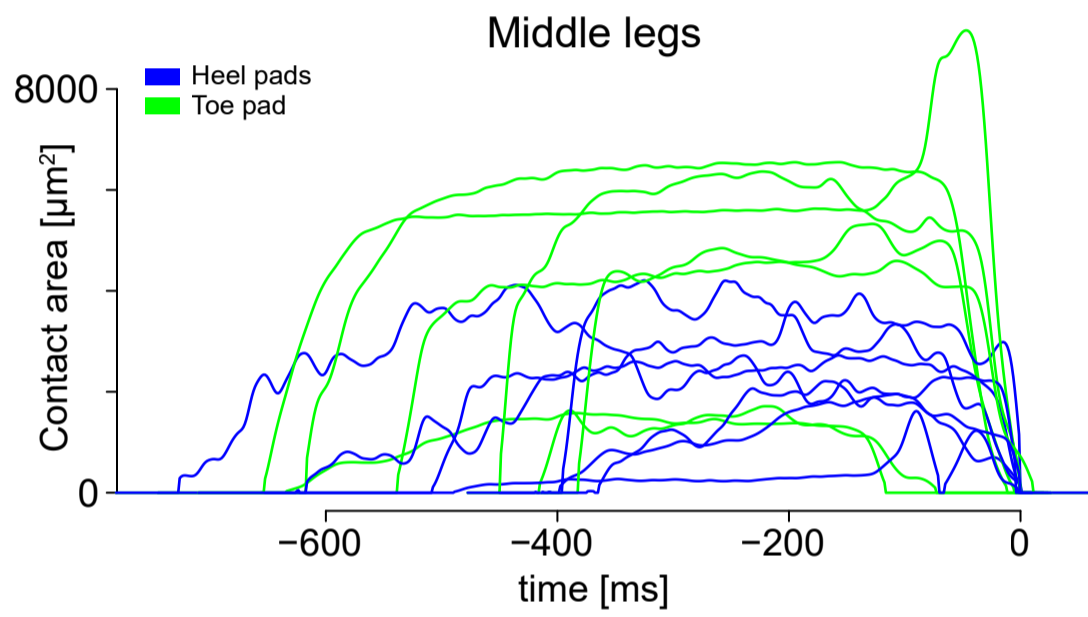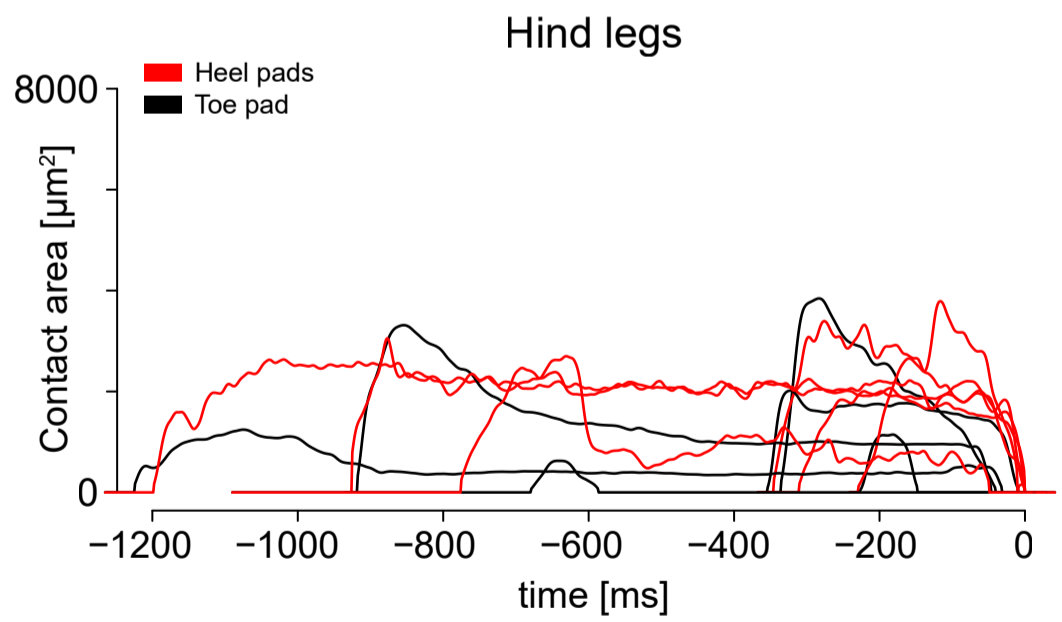

Supplement: Supplementary file 1 [file biomimetics-10-00069-s001.zip › FigS4-rev2.pdf]
